# Unfolding/Refolding Study on Collagen from Sea Cucumber Based on 2D Fourier Transform Infrared Spectroscopy

**DOI:** 10.3390/molecules21111546

**Published:** 2016-11-16

**Authors:** Lei Qin, Jing-Ran Bi, Dong-Mei Li, Meng Dong, Zi-Yuan Zhao, Xiu-Ping Dong, Da-Yong Zhou, Bei-Wei Zhu

**Affiliations:** 1 School of Food Science and Technology, Dalian Polytechnic University, Dalian 116034, China; qinlei@dlpu.edu.cn (L.Q.); bijingran125@163.com (J.-R.B.); i_Dongmeng@163.com (M.D.); 1201080407@cau.edu.cn (Z.-Y.Z.); dxiuping@163.com (X.-P.D.); zdyzf1@163.com (D.-Y.Z.); 2 National Engineering Research Center of Seafood, Dalian 116034, China; 3 School of Food and Biological Engineering, Jiangsu University, Zhenjiang 212013, China

**Keywords:** insoluble collagen fibrils, pepsin-solubilized collagens, 2D Fourier transform infrared spectrometry, thermostability, secondary structures

## Abstract

We aimed to explore the differences of thermal behaviors between insoluble collagen fibrils (ICFs) and pepsin-solubilized collagens (PSCs) from sea cucumber *Stichopus japonicus*. The unfolding/refolding sequences of secondary structures of ICFs and PSCs during the heating and cooling cycle (5 → 70 → 5 °C) were identified by Fourier transform infrared spectrometry combined with curve-fitting and 2D correlation techniques. ICFs showed a higher proportion of α-helical structures and higher thermostability than PSCs, and thus had more-stable triple helical structures. The sequences of changes affecting the secondary structures during heating were essentially the same between ICFs and PSCs. In all cases, α-helix structure was the most important conformation and it disappeared to form a β-sheet structure. In the cooling cycle, ICFs showed a partially refolding ability, and the proportion of β-sheet structure rose before the increasing proportion of α-helix structure. PSCs did not obviously refold during the cooling stage.

## 1. Introduction

The body walls of the sea cucumber *Stichopus japonicus* are enjoyed in Japan and China as a highly-priced delicacy, owing to their unique elasticity and palatability [1]. The dermis of the body wall is a typical connective tissue with mutable mechanical properties that rapidly changes in response to various stimuli [2]. Although the body walls of uncooked sea cucumbers are very hard to masticate, they become as tender as jelly after cooking [3]. Insoluble collagen fibrils (ICFs), the most abundant structural element in the dermis, account for about 70% of the total body wall proteins and are arranged into interwoven bundles [1,4]. The structural and rheological changes observed during cooking are due to denaturation and gelatination of collagen [5,6]. The unique textural properties of cooked sea cucumber are most probably attributable to the thermal denaturation of ICFs [1].

Collagen is a major structural protein composing the extracellular matrix of many tissues. Much progress has been made in elucidating the structure of collagen triple helices and the physicochemical basis for their stability [7,8]. However, the large size, insolubility, repetitive sequence and complex hierarchical structure of native collagen have thwarted most biochemical and biophysical analyses [8]. Pepsin digestion which removes the cross-linkages in the telopeptide region is always used to improve collagen solubility. So far, research about the biochemical and biophysical properties of sea cucumber collagen is mostly based on the pepsin-solubilized collagens (PSCs) [3,9,10,11]. It should be noted that the intermolecular cross-links are dominated by telopeptide domains, but after removal of terminal telopeptides, PSCs are not fully monomeric in solution and do not represent the native structure of dermic collagen.

To measure the effects of hierarchical structures on the thermal denaturation properties of sea cucumber collagen, we must devise and use suitable analytical and characterization methods. Fourier transform infrared spectroscopy (FTIR) is a useful method to monitor changes in the secondary structures of proteins [12,13,14]. Compared with ultraviolet-visible, nuclear magnetic resonance, circular dichroism, Raman spectroscopy, fluorescence, and X-ray crystallography which have often been used for the same purpose, high-quality FTIR spectra can be obtained with relative ease while preserving the solid, crystal and solution states of both native and denatured proteins [15].

In this study, naturally-crosslinked ICFs were extracted by removing mucopolysaccharide and soluble proteins, while PSCs were extracted by the pepsin digestion of ICFs. The purpose of this study was to explore and compare the thermal behaviors of PSCs and ICFs. The secondary structures of PSCs and ICFs were observed via FTIR. Different unfolding/refolding patterns of ICFs and PSCs were expounded by different FTIR data analysis methods during the heating and cooling cycle (5 → 70 → 5 °C).

## 2. Results and Discussion

### 2.1. Amino Acid Compositions of ICFs and PSCs

Table 1 lists the amino acid compositions of ICFs and PSCs. In general, the compositional characteristics are consistent with those reported in a previous study [3]. The high contents of characteristic amino acids, including glycine and hydroxyproline, suggest the homogeneity of ICFs and PSCs.

### 2.2. Denaturation Temperature of Sea Cucumber Collagen

**Figure 1:**
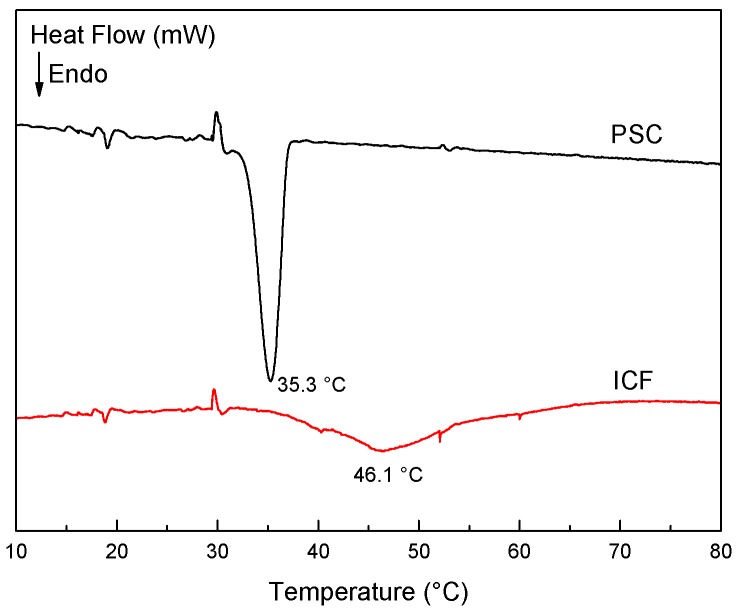
Comparison of DSC heating traces between aqueous 5% dispersions of ICFs (red line) and PSCs (black line).

Differential scanning calorimetry (DSC) is a well-developed analytical tool for the measurement of collagen transition, which is interpreted as the disintegration of triple helical structures into random coils, accompanied by heat absorption. Figure 1 shows the DSC scans of ICFs and PSCs dispersed in deionized water (5%, *w*/*v*). The maximum transition temperatures (T_max_) of PSCs is 10.8 °C lower than that of ICFs (35.3 vs. 46.1 °C), which suggests the triple helical structures in PSCs are less stable than those in ICFs. There are some important features that are noteworthy in comparing the thermal stability of IFCs and PSCs. Pepsin digestion can remove the cross-linkages in the telopeptide region of ICFs. PSCs, as a chain structure, were extracted by the pepsin digestion of ICFs [9]. Inter and intramolecular cross-links in ICFs may not only resulting in insoluble structures but also resulting in a higher transition temperature compared to PSCs.

### 2.3. FTIR Spectra of ICFs and PSCs

Considering the strong infrared absorption of H_2_O at the amide I region, we used D_2_O as the dispersion solution in FTIR analysis [13].

**Figure 2:**
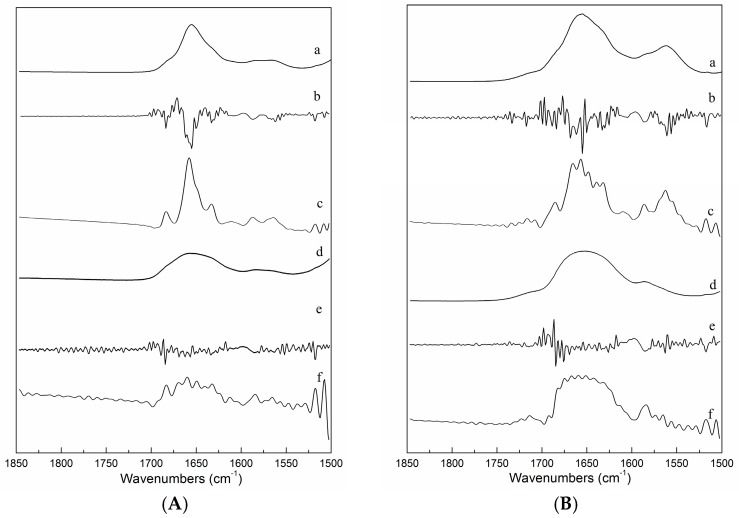
IR spectra of ICFs (**A**-**a**: original spectra at 5 °C; **A**-**b**: second derivative spectra at 5 °C; **A**-**c**: FSD spectra at 5 °C; **A**-**d**: original spectra at 50 °C; **A**-**e**: second derivative spectra at 50 °C; **A**-**f**: Fourier self-deconvolved spectra at 50 °C) and PSCs (**B**-**a**: original spectra at 5 °C; **B**-**b**: second derivative spectra at 5 °C; **B**-**c**: FSD spectra at 5 °C; **B**-**d**: original spectra at 50 °C; **B**-**e**: second derivative spectra at 50 °C; **B**-**f**: Fourier self-deconvolved spectra at 50 °C).

Figure 2A gives second derivative spectra and Fourier self-deconvolution (FSD) spectra for ICFs at 5 °C and 50 °C and the same set of spectra are given for PSCs in Figure 2B. The spectral changes detected by the FSD analyses were in agreement with those observed by the second derivative analysis. Prystupa and Donald [16] reported that the spectra obtained from D_2_O solutions were of better quality than those obtained for H_2_O solutions. 

**Figure 3:**
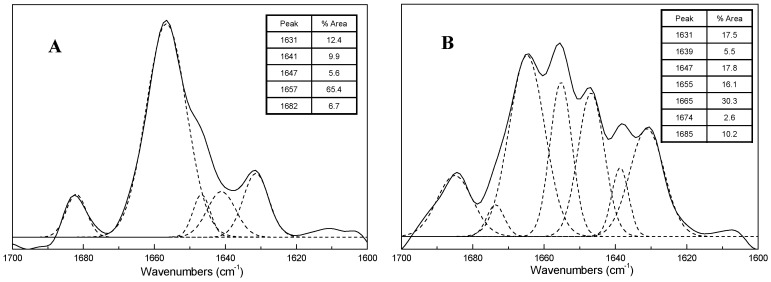
Fourier self-deconvolved infrared spectra in the amide I region of ICFs (**A**) and PSCs (**B**) obtained at 5 °C with the resolved underlying bands as deduced through peak-fitting analysis (5% *w*/*v* in D_2_O). The accompanying tables list the positions and relative percent areas of these components.

Figure 3 shows the amide I region in the FSD spectra of ICFs and PSCs dispersed in D_2_O (5% *w*/*v*) at 5 °C. The spectra were resolved to the underlying bands by a combination of FSD spectra and peak-fitting. The well-established empirical structure-frequency correlations (Table 2) indicate that β-sheet has a strong absorption band near 1631 cm^−1^ and a weaker band at high frequency (>1680 cm^−1^), whereas β-turn is reported at frequency near 1663–1671 cm^−1^. The peaks for α-helix and 3_10_-helix are located at ~1653 and ~1641 cm^−1^, respectively. Random coil structure is generally assigned to the band around 1645 cm^−1^.

In the present study, the amide I band absorption spectral profiles of ICFs and PSCs are evidently different. Clearly, at 5 °C, the ICFs spectrum exhibits five components (Figure 3A) while PSCs spectrum exhibits seven components (Figure 3B). The multiple structures of the collagen amide I are due to the heterogeneity of its peptide C=O group in a triple helix, a factor that directly influences the profile of the amide I band FTIR spectrum [17]. The major component of ICFs appears at ~1657 cm^−1^ (65.4% area), which means ICFs has predominant α-helical structure. In comparison, PSCs contain more β-turn (~1665 cm^−1^, 30.3%) and random coil (~1647 cm^−1^, 17.8%) than α-helix (~1655 cm^−1^, 16.1%). Collagen triple helices naturally aggregate to form fibrils and fibre bundles, which are stabilized by inter- and intramolecular cross-links, resulting in insoluble structures with higher molecular weight and thermostability [18,19,20].

### 2.4. FTIR Spectra as a Function of Temperature during Heating and Cooling of ICFs or PSCs

**Figure 4:**
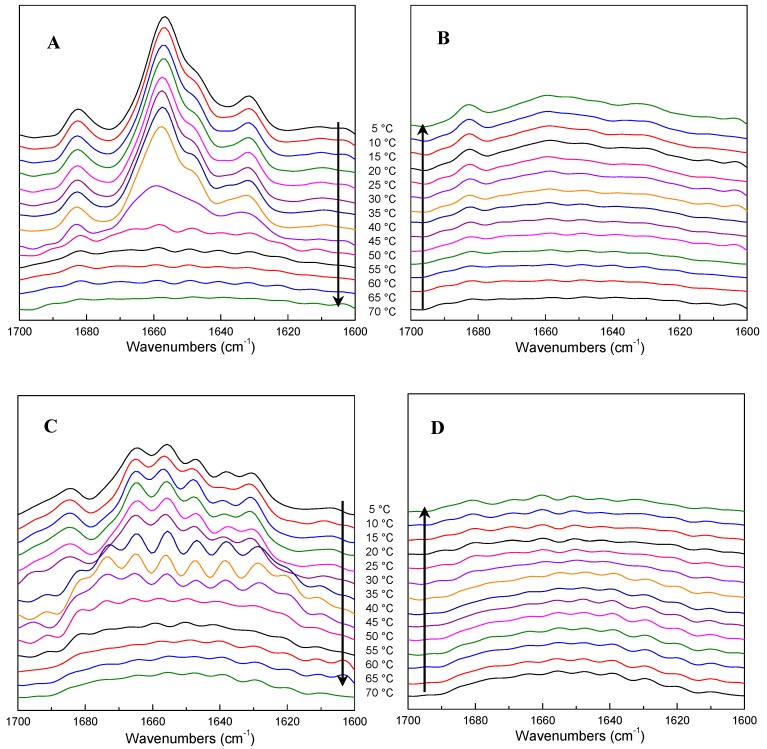
Fourier self-deconvolved infrared spectra in the amide I region of ICFs (**A**: heating; **B**: cooling) and PSCs (**C**: heating; **D**: cooling) (5% *w*/*v* in D_2_O).

The changes in the Fourier self-deconvolved infrared spectra in the amide I region upon heating (from 5 °C to 70 °C) and cooling (from 70 °C to 5 °C) of ICFs or PSCs dispersed in D_2_O (5% *w*/*v*) are shown in Figure 4.

As shown in Figure 4A, ICFs remained unchanged up to 40 °C, but were tremendously modified above 45 °C, corresponding to the transition temperature obtained by DSC (Figure 1). More marked changes in ICFs took place since 50 °C, which seemed a complete loss of α-helical structure (~1657 cm^−1^) relative to the α-helix content at 5 °C. In parallel, the bands at ~1631 and ~1682 cm^−1^ gradually decrease during the heating, but they change in the opposite way during the cooling (Figure 4B), as a slight increase occurs at ~1682, ~1657 and ~1631 cm^−1^.

The spectrum of PSCs shows no obvious differences until up to 35 °C (Figure 4C). At 35 °C, a new shoulder appears at ~1673 cm^−1^. Meanwhile, the bands at ~1647, ~1638 and ~1629 cm^−1^ all gradually increase. The total intensity of the amide I band begins to decrease at 45 °C, while the shoulder at ~1673 cm^−1^ vanishes gradually and the spectral shape becomes flat. During the cooling, no clear changes are revealed until down to 30 °C, while the peaks around ~1681, ~1669, ~1660, ~1651, ~1643, ~1632 cm^−1^ change subtly (Figure 4D).

The peaks both in the spectra of ICFs and PSCs at around 1656 cm^−1^ are characteristic of the amide I band between 1700 and 1600 cm^−1^. It was suggested that accurate prediction of the amide I band could be contoured from the three-dimensional structure of a protein [21]. ICFs and PSCs from the sea cucumber belong to type I collagen [3,9], which is constituted dominantly by the heterotrimers of two α_1_ (I) and one α_2_ (I) chains [7]. Bryan et al. [22] studied the thermal transition of a collagen model peptide by FTIR, they find that during denaturation, the band at 1645 cm^−1^ significantly decreased in intensity as temperature is increased, while the 1629 cm-1 band showed small changes, and the high wavenumber band (1667 cm^−1^) appears to broaden and disappear into the underlying broad feature. Our results showed that the intensity of the amide I band diminished and rebounded in the heating and cooling processes. These results revealed that the liberations might be responsible for weakening, breaking, re-forming and strengthening of the hydrogen bonds [23]. The results here are really based on the hypothesis that the amide I excitation energy strongly depends on the length and orientation of associated hydrogen bonds [24].

### 2.5. 2D Correlation Analysis of ICFs and PSCs

**Figure 5:**
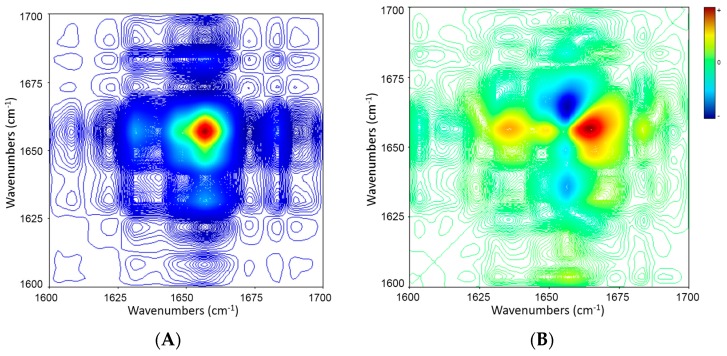
2D IR synchronous (**A**) and asynchronous (**B**) maps of amide I bands of ICFs (5% *w*/*v* in D_2_O) during heating (5 → 70 °C, in increment of 5 °C). Red and blue represent positive and negative correlation peaks, respectively.

The one-dimensional analysis was further extended to get more information on the sequence of structural events by 2D COS. Figure 5 displays 2D synchronous and asynchronous correlation maps of whole ICFs constructed from temperature-dependent spectral variations of heating process (from 5 to 70 °C) in the amide I region (1700–1600 cm^−1^). The synchronous correlation map (Figure 5A) shows a prominent auto-peak (at the diagonal) at 1656 cm^−1^ and two small cross-peaks (off the diagonal) at 1656/1631 and 1683/1656 cm^−1^. The asynchronous correlation map (Figure 5B) shows a positive cross-peak at 1683/1656 cm^−1^ and a negative one at 1656/1631 cm^−1^. A combined analysis of the signs in both synchronous and asynchronous maps in Table 3 reveals that the decrease in proportion of α-helix structures (1656 cm^−1^) happens after the decline in the proportions of β-sheet and β-turn structures (1631 and 1683 cm^−1^) in ICFs during the heating.

**Figure 6:**
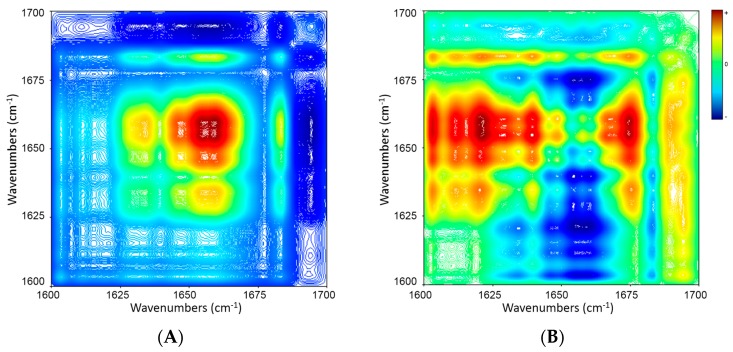
2D IR synchronous (**A**) and asynchronous (**B**) maps of amide I bands of ICFs (5% *w*/*v* in D_2_O) during cooling (70 → 5 °C, in decrement of 5 °C). Red and blue color represent positive and negative correlation peaks, respectively.

Figure 6 illustrates synchronous and asynchronous correlation maps of ICFs during the cooling from 70 to 5 °C. The synchronous map (Figure 6A) reveals four evident auto-peaks at 1634, 1646, 1657 and 1683 cm^−1^, respectively. The positive cross-peaks at 1683/1657, 1683/1646, 1683/1634, 1657/1646, 1657/1634 and 1646/1634 indicate that all these bands increase with temperature reduction. These results are well consistent with the 1D IR spectrum of Figure 4B. In addition, there are some negative cross-peaks (1634, 1646, 1657 and 1683 vs. 1695 cm^−1^). The asynchronous map (Figure 6B) shows positive cross-peaks at 1695 vs. 1611, 1634, 1657 and 1683 cm^−1^, and negative peaks at 1683 vs. 1611, 1634, 1646 and 1657 cm^−1^; 1657 vs. 1611 cm^−1^; 1646 vs. 1611 cm^−1^; and 1634 vs. 1611 cm^−1^. The sequences of band intensity changes based on the signs of the cross-peaks in the synchronous and asynchronous maps are summarized in Table 4. ICFs experienced the following sequence of spectral changes during cooling: (1) increase in proportion of β-sheet structure (1634 cm^−1^); (2) increase in proportion of random coil structure (1646 cm^−1^); (3) increase in proportion of α-helix structure (1657 cm^−1^); (4) increase in proportion of β-turn structure (1683 cm^−1^); (5) decrease in proportion of β-turn structure (1695 cm^−1^). This sequence is contrary to the result in the heating process.

**Figure 7:**
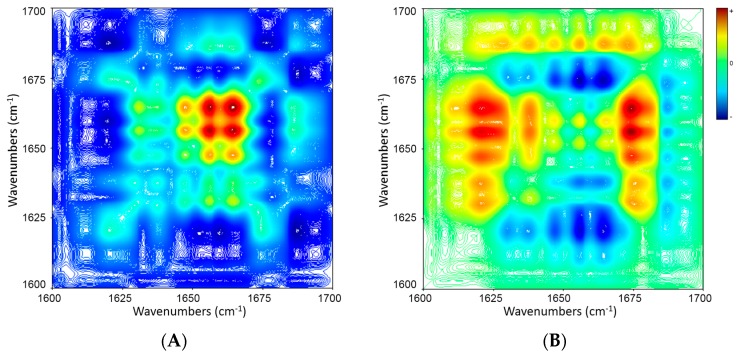
2D IR synchronous (**A**) and asynchronous (**B**) maps of amide I bands of PSCs (5% *w*/*v* in D_2_O) during heating (5 → 70 °C, in increment of 5 °C). Red and blue color represent positive and negative correlation peaks, respectively.

Figure 7 shows synchronous and asynchronous correlation spectra of PSCs during heating. The band at 1621 cm^−1^ assigned to β-sheet structure was not considered owing to its fluctuation with temperature rise, which complicated the interpretation of the 2D COS results. The synchronous map (Figure 7A) shows two strong auto-peaks (1656 and 1664 cm^−1^) and five weak auto-peaks (1630, 1637, 1648, 1673 and 1686 cm^−1^). Positive cross-peaks appear at 1637, 1648, 1656, 1664 vs. 1630 cm^−1^; 1648, 1656, 1664, 1673 vs. 1637 cm^−1^; 1656, 1664, 1686 vs. 1648 cm^−1^; 1664, 1686 vs. 1656 cm^−1^; 1686 vs. 1664 cm^−1^ while there is no negative cross-peak. The asynchronous map (Figure 7B) shows positive cross-peaks at 1673 vs. 1637, 1648, 1656, 1664 cm^−1^ and negative cross-peaks at 1686 vs. 1637, 1648, 1656, 1664, 1673 cm^−1^; 1664 vs. 1637 cm^−1^; 1656 vs. 1637 cm^−1^; 1648 vs. 1637 cm^−1^. The sequences of band intensity changes based on the signs of the cross-peaks in the synchronous and asynchronous maps are summarized in Table 5. PSCs experienced the following sequence of spectral changes during the heating: (1) decrease in proportion of β-turn structure (1673 cm^−1^); (2) decrease in proportion of β-sheet structure (1637 and 1630 cm^−1^); (3) decrease in proportions of random coil structure (1648 cm^−1^), α-helix structure (1656 cm^−1^) and β-turn structure (1664 cm^−1^); (4) decrease in proportion of β-turn structure (1686 cm^−1^).

**Figure 8:**
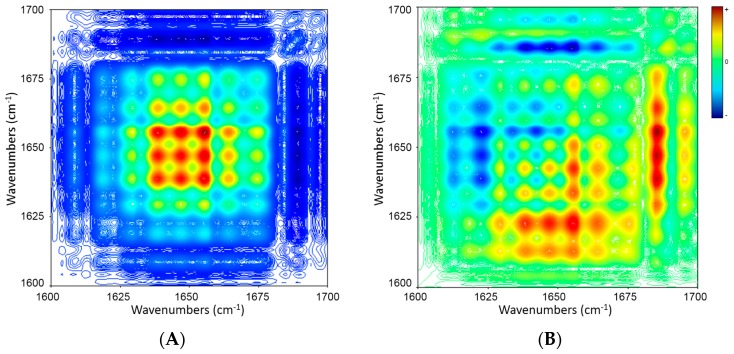
2D IR synchronous (**A**) and asynchronous (**B**) maps of amide I bands of PSCs (5% *w*/*v* in D_2_O) during cooling (70 → 5 °C, in decrement of 5 °C). Red and blue color represent positive and negative correlation peaks, respectively.

Figure 8 displays synchronous and asynchronous correlation spectra of PSCs during cooling. The synchronous map shows three strong auto-peaks (1639, 1647 and 1656 cm^−1^) and four weak auto-peaks (1629, 1664, 1675 and 1689 cm^−1^) (Figure 8A). The sequences of band intensity changes based on the signs of the cross-peaks in the synchronous and asynchronous correlation maps are summarized in Table 6.

A combined analysis of the signs in both synchronous and asynchronous maps [25] reveals the following sequence of events during cooling: (1) increase in proportion of β-turn structure (1664 cm^−1^); (2) increase in proportion of α-helix structure (1656 cm^−1^); (3) increase in proportions of β-turn structure (1629 cm^−1^), 3_10_-helix structure (1639 cm^−1^), β-sheet structure (1675 cm^−1^) and random coil structure (1647 cm^−1^); (4) increase in proportion of β-sheet structure (1618 cm^−1^); (5) decrease in proportion of β-turn structure (1689 cm^−1^).

As a whole, the α-helix and β-sheet structures of both ICFs and PSCs are quite sensitive to temperature changes. The α-helix is driven especially by the formation of hydrogen bond network along the polypeptide backbone [26]. The β-sheet structure was derived from the α-helical structures [27]. Our study shows that along with the temperature rise, the proportion of α-helix diminishes with the increasing proportion of β-sheet structure, and the changes of β-sheet always occur before those of α-helix. These results agree well with previous research [28,29], indicating the collagen-unfold structures were formed during the heating process. In a collagen-unfold structure, the aligned α-helical domain is divided into single chains, which suggests that the regenerative β-sheet is the intermediate instead of single chains and is very unstable [16,28]. As a consequence, the partial β-sheet bands in ICFs disappeared during the cooling, which indicate a partial reverse of the collagen-fold structures [16]. However, ICFs and PSCs showed some differences. The denaturation temperature of ICFs (45.1 °C) was 10.8 °C higher than that of PSCs, which was an earlier disappearing signal of the triple helical structure in the event sequence of PSCs. In addition, ICFs had a partial refolding ability during the cooling, while was not observed in PSCs. Inter and intramolecular cross-links in ICFs may have an important effect for the thermal stability and the refolding ability during the cooling process.

## 3. Materials and Methods

### 3.1. Materials and Chemicals

Live specimens of sea cucumber *S. japonicus* were collected from Lvshun sea coast, Dalian, China. After being washed in deionized water, the body walls were dissected, treated by removal of all the adherent tissues with tweezers and then stored at −80 °C until used. Pepsin was purchased from Sangon Biotech Co. (Shanghai, China). Deuterium oxide (D_2_O) was provided by Beijing Chongxi Science and Technology Incubator Co. (Beijing, China). All other reagents used here were analytical grade and acquired from commercial vendors.

### 3.2. Extraction of ICFs and PSCs from Sea Cucumber Body Walls

ICFs and PSCs were isolated from the body walls of sea cucumber as described by previous study [3]. Briefly, ICFs were extracted from 100 g of minced tissue pieces that had been extensively washed with deionized water. After centrifugation at 13,680× *g* for 10 min, the materials were added with 1 L of 0.1 M Tris–HCl buffer (pH 8.0, 5 mM ethylene diamine tetraacetic acid (EDTA), 0.5 M NaCl) for precipitation overnight under stirring. After centrifugation at 13,680× *g* for 10 min, the precipitates were collected, mixed with 1 L of deionized water and stirred for 48 h. After centrifugation at 9000× *g* for 5 min, the supernatant containing free collagen fibrils were collected and the pellets were washed with another 500 mL of deionized water. After the combined supernatants were centrifuged at 17,300× *g* for 30 min, the resultant precipitates were stirred with 0.1 M NaOH to remove non-collagenous substance, which minimized the effects of endogenous proteinases on collagen fibrils. Then the precipitates of collagen fibrils were washed with deionized water and lyophilized to get ICFs.

For preparation of PSCs, first the ICFs were digested with pepsin at 4 °C for 72 h. Then the digested solution was centrifuged at 17,300× *g* for 20 min and PSCs in the supernatant were salted out by adding NaCl to a final concentration of 0.8 M. After centrifugation at 13,600× *g* for 5 min, the precipitates were collected and dissolved in 0.5 M acetic acid. The PSCs solution was dialyzed in a Na_2_HPO_4_–NaH_2_PO_4_ buffer (0.02 M, pH 8.0) for 48 h to deactivate the pepsin. Finally, after dialysis against 0.1 M acetic acid for 72 h, the retentate was dialyzed against deionized water to transfer acetic acid entirely and then lyophilized to form PSCs.

### 3.3. Amino Acid Composition Analysis

The amino acid compositions of ICFs and PSCs were analyzed after hydrolysis with 6 M hydrochloric acid at 110 °C for 24 h and then derivatization with 2,4-dinitrofluorobenzene. Amino acid levels were determined by LC-10Avp Plus high-performance liquid chromatograph (HPLC; Shimadzu Co., Tokyo, Japan) coupled with an amino acid analysis column (Elite Analytical Instruments Co., Ltd., Dalian, China). Condition setting and derivatization were performed according to the manufacturer’s instruction (Elite Analytical Instruments Co., Ltd.). The amino acids were quantified on LCsolution 1.11 SP1 (Shimadzu Co.) based on peak areas of known concentrations of the standards.

### 3.4. Thermal Analysis of ICFs and PSCs

The maximum temperatures of endothermic transition (T_max_) for ICFs and PSCs were measured by a microSC differential scanning microcalorimeter (SETARAM Instrumentation, Caluire, France). The samples were first cooled from room temperature to 4 °C and then heated to 80 °C at a rate of 1 °C/min. All samples were tested in triplicate.

### 3.5. FTIR Measurements

To determine the effects of thermal treatment on the secondary structures of ICFs and PSCs, we re-suspended the samples in D_2_O, which was used to study the amide I region (1700–1600 cm^−1^) without any H_2_O contribution [30].

FTIR spectra were collected on an FTIR spectrometer (Perkin Elmer Corporation, Norwalk, CT, USA), which was continuously purged by dry nitrogen during the data collection. Each sample suspension (5% *w*/*v*) was placed in a heatable IR transmission cell between two CaF_2_ windows separated by a 50 µm teflon spacer [31]. The cell temperature was regulated by a high-precision constant temperature system (Ningbo Scientz Biotechnology, Ningbo, China). The temperature change from 5 to 70 °C was obtained by successive heating/cooling cycles at a rate of 1 °C/min with an instrument equilibration time of 10 min before the spectral recording [22,32]. To ensure the signal-to-noise ratio was acceptable, we determined each spectrum by co-addition of 5 scans at a 2 cm^−1^ resolution.

The spectral interval of 1700–1600 cm^−1^ defined as the amide I region was dealt with baseline interaction corrections at 1700 and 1600 cm^−1^. The data were smoothed on Omnic 6.0 (Thermo Electron Corporation, Madison, WI, USA) at the point of 5. Fourier self-deconvolution in this region was carried out at a bandwidth of 36.5 cm^−1^ and a resolution enhancement of 2.6 [12,13]. The band assignment in the amide I region is listed in Table 2.

### 3.6. Curve-Fitting

Quantitative values for areas of heavily-overlapped bands were achieved by curve-fitting [33]. The number and positions of bands in which curves were fitted in the FSD spectra were determined on *Origin 8.5* (OriginLab Corp., Northampton, MA, USA). The spectra were curve-fitted with Gaussian band profiles [34,35].

### 3.7. 2D Correlation Analysis

IR two-dimensional correlation spectroscopy (2D COS) was performed by IR 2D-COS software (designed by Tsinghua University Analysis Center, Beijing, China). Synchronous and asynchronous correlation intensities were computed from the spectra recorded as a function of temperature rise or drop [36].

Synchronous maps consist of two types of peaks: (1) auto-peaks, on the diagonal, which are always positive and indicative of temperature-induced changes in band intensity; (2) cross-peaks, at the off-diagonal, which indicate the band intensity changes at the corresponding wave numbers on the x- and y-axes are correlated. A positive cross-peak indicates that the intensities of two bands change in the same direction (e.g., both bands both increase or both decrease with temperature rise), and vice versa. Asynchronous maps do not show auto-peaks, and the presence of cross-peaks indicates that changes in the correlated bands take place out-of-phase, accelerated or delayed with respect to each other. A positive sign of the synchronous and asynchronous cross-peaks at wave numbers x = ν1 and y = ν2 indicates that the intensity change at ν1 occurs prior to that at ν2. If the change at ν1 takes place after that at ν2, the sign of the cross-peak on the asynchronous map is negative. If the cross-peak on the synchronous map is negative, the sign convention is reversed. Thus, the sequence of events could be established by comparing the signs of cross-peaks in the synchronous and asynchronous maps [25].

## 4. Conclusions

FTIR combined with curve-fitting was used to analyze the secondary structures of ICFs and PSCs. ICFs showed a higher proportion of α-helical structure than PSCs. The denaturation temperatures of ICFs and PSCs were 46.1 °C and 35.3 °C respectively, which suggested the higher thermostability of ICFs. The 2D correlation analysis showed the sequences of changes affecting the secondary structure during heating were essentially the same between ICFs and PSCs. In all cases, α-helix structure was the most important conformation and disappeared with the formation of β-sheet structures. In the cooling cycle, ICFs showed a partial refolding ability, and the proportion of β-sheet structure increased before the proportion of α-helix structure rose. However, PSCs did not show visible refolding during the cooling process. ICFs were mainly stabilized by inter- and intramolecular cross-links, resulting in insoluble structures and higher thermostability.

## Figures and Tables

**Table 1:** Amino acid compositions of ICFs and PSCs from the body walls of *Stichopus japonicas* (residues/100).

Amino Acid	ICF	PSC
Aspartic acid	6.57 ± 0.08	6.66 ± 0.03
Glutamic acid	10.71 ± 0.05	10.66 ± 0.08
Hydroxyproline	6.06 ± 0.10	6.13 ± 0.03
Serine	4.61 ± 0.03	4.60 ± 0.04
Arginine	5.71 ± 1.61	3.69 ± 1.81
Glycine	34.05 ± 0.95	36.44 ± 1.72
Threonine	3.87 ± 0.04	3.88 ± 0.02
Proline	9.02 ± 0.02	8.98 ± 0.03
Alanine	7.07 ± 0.02	7.03 ± 0.02
Valine	2.24 ± 0.05	2.15 ± 0.06
Methionine	0.70 ± 0.14	0.51 ± 0.47
Isoleucine	1.74 ± 0.02	1.72 ± 0.03
Leucine	1.92 ± 0.03	1.88 ± 0.03
Phenylalanine	0.76 ± 0.11	1.89 ± 0.60
Histidine	3.02 ± 0.40	2.08 ± 0.80
Lysine	0.85 ± 0.01	0.77 ± 0.06
Tyrosine	1.11 ± 0.18	0.91 ± 0.11

**Table 2:** Deconvoluted amide I band frequencies and assignments to protein secondary structure in D_2_O [13].

Wavenumber (cm^−1^)	Assignment
1624 ± 4	β-sheet
1631 ± 3	β-sheet
1637 ± 3	β-sheet
1641 ± 2	3_10_-helix
1645 ± 4	random coil
1653 ± 4	α-helix
1663 ± 4	β-turn
1671 ± 3	β-turn
1675 ± 5	β-sheet
1683 ± 2	β-turn
1689 ± 2	β-turn
1694 ± 2	β-turn

**Table 3:** Correlation table for deconvolved FTIR spectra of CCFs (5% *w*/*v* in D_2_O) during heating (5 → 70 °C, in increment of 5 °C).

Wavenumber (cm^−1^)	1683 ↓	1656 ↓
1631 ↓	≈	+ − →
1656 ↓	+ + ←	

Column and row headings represent the x and y wavenumbers, respectively, from the 2D maps in Figure 5. Vertical arrows represent an increase (↑) or decrease (↓) in the band intensity as the temperature increased, as deduced from 1D spectra. The signs “+” and “–“ indicated positive and negative peaks in the 2D synchronous map and asynchronous map, respectively. The sign “≈” indicated there is no correlation peak is found in the asynchronous map which means both peaks change at the same time. Horizontal arrows indicate that the intensity change at y·cm^−1^ occurs before (→) or after (←) that at x·cm^−1^.

**Table 4:** Correlation table for deconvolved FTIR spectra of ICFs (5% *w*/*v* in D_2_O) upon cooling (70 → 5 °C, in decrement of 5 °C).

Wavenumber (cm^−1^)	1695 ↓	1683 ↑	1657 ↑	1646 ↑	1634 ↑
1611 ↑	− + →	+ − →	+ − →	+ − →	+ − →
1634 ↑	− + →	+ − →	+ − →	+ − →	
1646 ↑	− + →	+ − →	+ − →		
1657 ↑	− + →	+ − →			
1683 ↑	− + →				

Column and row headings represent the x and y wavenumbers, respectively, from the 2D maps in Figure 6. Vertical arrows represent an increase (↑) or decrease (↓) in the band intensity as the temperature increased, as deduced from 1D spectra. The signs “+” and “–“ indicated positive and negative peaks in the 2D synchronous map and asynchronous map, respectively. The sign “≈” indicated there is no correlation peak is found in the asynchronous map which means both peaks change at the same time. Horizontal arrows indicate that the intensity change at y·cm^−1^ occurs before (→) or after (←) that at x·cm^−1^.

**Table 5:** Correlation table for deconvolved FTIR spectra of PSCs (5% *w*/*v* in D_2_O) upon heating (5 → 70 °C, in increment of 5 °C) and the sequence of structural changing events.

Wavenumber (cm^−1^)	1686 ↓	1673 ↓	1664 ↓	1656 ↓	1648 ↓	1637 ↓
1630 ↓	+ − →	+ + ←	+ − →	+ − →	+ − →	≈
1637 ↓	+ − →	+ + ←	+ − →	+ − →	+ − →	
1648 ↓	+ − →	+ + ←	≈	≈		
1656 ↓	+ − →	+ + ←	+ − →			
1664 ↓	+ − →	+ + ←				
1673 ↓	+ − →					

Column and row headings represent the x and y wavenumbers, respectively, from the 2D maps in Figure 7. Vertical arrows represent an increase (↑) or decrease (↓) in the band intensity as the temperature increased, as deduced from 1D spectra. The signs “+” and “–“ indicated positive and negative peaks in the 2D synchronous map and asynchronous map, respectively. The sign “≈” indicated there is no correlation peak is found in the asynchronous map which means both peaks change at the same time. Horizontal arrows indicate that the intensity change at y·cm^−1^ occurs before (→) or after (←) that at x·cm^−1^.

**Table 6:** Correlation table for deconvolved FTIR spectra of PSCs (5% *w*/*v* in D_2_O) upon cooling (70 → 5 °C, in increment of 5 °C) and the sequence of structural changing events.

Wavenumber (cm^−1^)	1689 ↓	1675 ↑	1664 ↑	1656 ↑	1647 ↑	1639 ↑	1629 ↑
1618 ↑	− + →	+ + ←	+ + ←	+ + ←	+ + ←	+ + ←	+ + ←
1629 ↑	− + →	+ + ←	+ + ←	+ + ←	+ + ←	≈	
1639 ↑	− + →	≈	+ + ←	+ + ←	≈		
1647 ↑	− + →	+ − →	+ + ←	+ + ←			
1656 ↑	− + →	+ − →	+ + ←				
1664 ↑	− + →	+ − →					
1675 ↑	− + →						

Column and row headings represent the x and y wavenumbers, respectively, from the 2D maps in Figure 8. Vertical arrows represent an increase (↑) or decrease (↓) in the band intensity as the temperature increased, as deduced from 1D spectra. The signs “+” and “–“ indicated positive and negative peaks in the 2D synchronous map and asynchronous map, respectively. The sign “≈” indicated there is no correlation peak is found in the asynchronous map which means both peaks change at the same time. Horizontal arrows indicate that the intensity change at y·cm^−1^ occurs before (→) or after (←) that at x·cm^−1^.
